# Implications of notch signaling in duchenne muscular dystrophy

**DOI:** 10.3389/fphys.2022.984373

**Published:** 2022-09-27

**Authors:** Lily Den Hartog, Atsushi Asakura

**Affiliations:** Department of Neurology, Stem Cell Institute, Paul and Sheila Wellstone Muscular Dystrophy Center, University of Minnesota Medical School, Minneapolis, MN, United States

**Keywords:** notch, muscular dystrophy, muscle regeneration, satellite cell, muscle stem cell

## Abstract

This review focuses upon the implications of the Notch signaling pathway in muscular dystrophies, particularly Duchenne muscular dystrophy (DMD): a pervasive and catastrophic condition concerned with skeletal muscle degeneration. Prior work has defined the pathogenesis of DMD, and several therapeutic approaches have been undertaken in order to regenerate skeletal muscle tissue and ameliorate the phenotype. There is presently no cure for DMD, but a promising avenue for novel therapies is inducing muscle regeneration via satellite cells (muscle stem cells). One specific target using this approach is the Notch signaling pathway. The canonical Notch signaling pathway has been well-characterized and it ultimately governs cell fate decision, cell proliferation, and induction of differentiation. Additionally, inhibition of the Notch signaling pathway has been directly implicated in the deficits seen with muscular dystrophies. Here, we explore the connection between the Notch signaling pathway and DMD, as well as how Notch signaling may be targeted to improve the muscle degeneration seen in muscular dystrophies.

## Introduction

Muscular dystrophies are a group of inherited disorders that involve progressive muscle weakness and degeneration of skeletal muscle (Lovering et al., 2005). Duchenne muscular dystrophy (DMD) is one of the most severe forms of inherited muscular dystrophy, proving to be lethal in 100% of cases, and is also the most prevalent, with an incidence of one per 5,136 male births (Crisafulli et al., 2020; Yao et al., 2021). This qualifies it as one of the most widespread recessive disorders among the human population (Mah, 2016). Becker muscular dystrophy (BMD) is also prevalent, but somewhat less common and less severe (Duan et al., 2021). Since there is currently no cure for DMD, it is crucial that further research be done in order to identify and optimize potential treatments and therapies (Guiraud et al., 2015).

DMD is caused by mutations of the *dystrophin*/*DMD* gene, which is located at the Xp21 locus on the X chromosome (Venugopal and Pavlakis, 2022). Because DMD is an X-linked disorder, it almost exclusively affects males, with females acting as asymptomatic carriers (Crisafulli et al., 2020). The *dystrophin* gene is the largest known human gene, containing 79 exons and spanning >2,200 kb (Gao and McNally, 2015). Secondary to its large size is its complex mutational spectrum; it has a high spontaneous mutation rate and there are >7,000 known mutations of the gene, with 1/3 of DMD cases resulting from sporadic mutations (Mah, 2016; Venugopal and Pavlakis, 2022). In current research, mutation rate is the probability that a unit length of DNA will mutate over time. Thus, the *dystrophin* gene has a high mutation rate, in part due to its huge gene size (Balin and Cascalho, 2010).

Deletion mutations account for 60%–70% of DMD cases, and point mutations and exonic duplications are also common (Gao and McNally, 2015; Duan et al., 2021). *Dystrophin* codes for the protein dystrophin, whose production can be limited and even eliminated by *dystrophin* mutation (Duan et al., 2021; Yao et al., 2021). Out-of-frame *dystrophin* mutations or premature stop-codon mutations typically lead to complete loss of the dystrophin protein and are more severe, while in-frame mutations that lead to the synthesis of a partially functional truncated protein produce milder forms of muscular dystrophy, such as BMD (Mah, 2016; Duan et al., 2021).

Dystrophin is located at the sarcolemma (cell membrane) of skeletal muscle cells and cardiomyocytes, interacting with a group of peripheral membrane and transmembrane proteins to form the dystrophin-associated glycoprotein complex (DGC) (Jiang et al., 2014). In healthy muscle, the DGC provides structural stability to skeletal and heart muscle, participates in transmembrane signaling, and plays a role in the vasomotor response to physical activity (Mah, 2016). In dystrophic muscle, however, loss of dystrophin diminishes the DGC, leading to weakness of the cytoskeleton, sarcolemmal lesions, and increased membrane fluidity (Wilschut et al., 2012; Venugopal and Pavlakis, 2022). This causes abnormal calcium influx and inflammation, which has catastrophic ramifications including the altered composition of structural glycoproteins in the extracellular matrix, activation of proteases and proinflammatory cytokines, ischemic injury, oxidative and nitrosative stress, and mitochondrial dysfunction (Duan et al., 2021). As a result of this loss in myofiber integrity, resident muscle stem cells undergo fibrogenesis rather than myogenesis, causing aberrant collagen deposition and subsequent necrosis (Wilschut et al., 2012; Kornegay, 2017). Through repeated cycles of necrosis and regeneration, muscle is gradually replaced with fibrous connective tissue and fat, producing the phenotypic characteristics of DMD (Venugopal and Pavlakis, 2022).

Skeletal muscle regeneration is carried out by the proliferation of quiescent muscle satellite cells (muscle stem cells) and the differentiation of myofibers. Activation of the Notch pathway is required to maintain the quiescent state of muscle satellite cells, and disruption of the Notch pathway leads to disruption of muscle satellite cell maintenance, thereby impairing muscle regeneration. Hence, disruption of the Notch pathway is one of the mechanisms of skeletal muscle disease. Currently, three muscle disease genes that interact with the Notch pathway have recently been identified—Jag2, MEGF10, and POGLUT1—whose mutation has been noted in muscular dystrophies. This review introduces the pathogenesis and treatment strategies for DMD, and discusses the biology of the Notch pathway and genetics in muscular dystrophies.

### Pathology of DMD

DMD symptoms occur as early as 2–3 years of age and the disorder causes death prior to the third or fourth decade of life, usually around ages 19–25 (Mah, 2016; Duan et al., 2021; Iftikhar et al., 2021). DMD initially presents with symptoms of difficulties climbing stairs, a waddling gait, toe walking, Gowers’ sign, and frequent falls (Birnkrant et al., 2018b; Duan et al., 2021). The physiological presentation of DMD goes on to primarily include progressive muscle degeneration, proximal weakness, and joint contractures (Mah, 2016; Coppens et al., 2021). Due to this, DMD patients are typically wheelchair-dependent by 10–12 years of age (Duan et al., 2021). DMD eventually brings about numerous secondary complications, including scoliosis, respiratory insufficiency, and cardiac issues (Mah, 2016). Dilated cardiomyopathy, myocardial necrosis, conduction defects, and arrhythmias are very common as well, with cardiorespiratory failure being the leading cause of mortality in DMD patients (Mah, 2016; Yao et al., 2021). This can be attributed to the absence of dystrophin in cardiomyocytes, which causes a loss of contractile function (Yucel et al., 2018). Additionally, though most DMD patients do not fit the criteria for intellectual disability, most do have some degree of cognitive impairment (Yucel et al., 2018). Many DMD patients do have a below-average IQ, as studies have reported that 20% of DMD patients have an IQ lower than 70 and 44% of DMD patients have learning disabilities (Yucel et al., 2018; Mohamadian et al., 2022). DMD patients also have been shown to be at increased risk for neurobehavioral comorbidities, such as attention-deficit/hyperactivity disorder, autism spectrum disorder, epilepsy, and anxiety (Mohamadian et al., 2022). There has been a suggestion of genotype-phenotype association with regards to *dystrophin* mutation and neurodevelopmental manifestations, as Ricotti et al. found that patients with distal mutations in the *dystrophin* gene were more likely to have neurodevelopmental problems, intellectual disability, memory deficits, and decreased grey matter volume. However, this study also demonstrated that emotional and behavioral problems were equally distributed among patients with proximal versus distal *dystrophin* mutations, calling into question this genotype-phenotype association (Ricotti et al., 2016). Cognitive impairments seen in DMD patients have typically been thought of as non-progressive, though recent studies have suggested otherwise (Bagdatlioglu et al., 2020). Studies in a mouse model of DMD have demonstrated that long-term memory and anxiety behaviors do worsen with age, which indicates that dystrophin deficiency causes progressive cognitive impairment greater than that naturally seen with aging, and furthermore that the reduced life expectancy of human DMD patients may mask their potential for progressive cognitive impairment (Bagdatlioglu et al., 2020).

DMD is medically evaluated in several manners, including *via* laboratory work, muscle biopsy, gene analysis, electromyography, and cardiac testing (Venugopal and Pavlakis, 2022). The possibility of newborn screening has been extensively discussed, and a recent survey revealed that most DMD physicians would see a benefit in newborn screening and feel that the DMD care community is ready for this (Armstrong et al., 2022). This may be of greatly advantageous, as the majority of DMD physicians also indicated that they would recommend initiating therapies much earlier than the typical age at which DMD is currently diagnosed (Armstrong et al., 2022). However, this has not yet become standard (Birnkrant et al., 2018b). The diagnosis of DMD is usually confirmed after symptoms are present via a laboratory test for serum creatine kinase (CK), a muscle enzyme that, when elevated, reflects ongoing muscle damage (Duan et al., 2021). Serum CK is often elevated in DMD patients before the development of clinical symptoms, as levels peak by age two and its level is typically 10–20x greater than the upper limit of normal (Venugopal and Pavlakis, 2022). Other muscle enzymes, including aldolase, alanine transaminase (ALT), and aspartate aminotransferase (AST), are often elevated as well, though not used as a diagnostic measure (Duan et al., 2021). Though serial muscle biopsies from DMD patients are not usually necessary for diagnosis, they do reveal multiple dystrophic changes, which include disorganization, scattered degeneration, connective tissue proliferation, inflammation, muscle fiber necrosis, and extensive deposits of adipose tissue in place of muscle (Guiraud et al., 2015; Coppens et al., 2021; Venugopal and Pavlakis, 2022). Gene analysis of DMD patients shows a complete absence of the *dystrophin* gene, and dystrophin immunoblotting may be utilized in order to predict the severity of the disease (Venugopal and Pavlakis, 2022). In examining muscles, electromyography can detect nonspecific myopathic features associated with muscular dystrophy (Venugopal and Pavlakis, 2022). Finally, cardiac testing is used to evaluate for complications frequently seen with DMD; electrocardiogram reveals characteristic changes, telemetry identifies conduction abnormalities, and echocardiogram shows evidence of dilated cardiomyopathy, which is present in nearly all DMD patients by the time they reach their twenties (Venugopal and Pavlakis, 2022).

## Current treatment for DMD

Despite its prevalence and severity, there is currently no cure for DMD and most available therapies simply act to manage symptoms and prolong ambulation and lifespan (Birnkrant et al., 2018b; Yao et al., 2021). As laid out clearly in a multi-part review by Birnkrant et al., a multidisciplinary approach to treatment is critical, typically led by a neuromuscular specialist. Corticosteroids and supportive measures have remained the standard of care over the past 30 years, since the molecular basis of DMD was defined (Motohashi et al., 2019). Corticosteroids, usually prednisone or deflazacort, are typically initiated around 4–5 years of age and provide numerous benefits for DMD patients, including deceleration of myofiber necrosis, improved pulmonary function, delayed development of scoliosis, prolonged independent ambulation, reduced progression of cardiomyopathy, improved mortality, and stabilization of muscle strength and function (Mah, 2016; Birnkrant et al., 2018b; Iftikhar et al., 2021; Venugopal and Pavlakis, 2022). That said, there is a slew of adverse side effects associated with corticosteroid use, including short stature, obesity, and cataracts, deeming it a suboptimal treatment (Mah, 2016). Corticosteroids also greatly increase the probability that DMD patients develop osteoporosis and skeletal fractures, including vertebral compression fractures, for which they are already at an elevated risk (Birnkrant et al., 2018a). Corticosteroid treatment is typically supplemented by supportive interventions that act to prolong survival and lessen symptom severity. To account for cardiac troubles, DMD patients are typically treated with angiotensin-converting enzyme (ACE) inhibitors and beta-blockers in order to slow the progression of cardiomyopathy and attempt to prevent heart failure (Venugopal and Pavlakis, 2022). It is recommended that spirometry be initiated when the patient is 5–6 years old, and noninvasive positive pressure ventilation and effective airway clearance are used to manage respiratory issues (Mah, 2016; Birnkrant et al., 2018a). Orthopedic management and physiotherapy are employed with the primary goals of minimizing joint contractures, maintaining a straight spine, and promoting bone health (Birnkrant et al., 2018a). Finally, endocrine and gastrointestinal management are also crucial to the appropriate treatment of DMD patients (Birnkrant et al., 2018b).

Recent scientific advances have garnered the potential for novel therapies to fight numerous neuromuscular diseases, including DMD. Currently, many therapeutic approaches aim to rescue dystrophin expression in skeletal muscle with hopes of improving function in dystrophic muscle, promoting muscle hypertrophy, and reducing muscle wasting (Cossu and Sampaolesi, 2007; Vieira et al., 2015). The goal of these therapies is not to cure DMD, but rather to improve severe DMD phenotypes to mimic more mild phenotypes, similar to BMD (Tominari and Aoki, 2022). A common limitation in the quest for effective DMD therapies is that there is no specific set of recommended or required outcome measures, making goals ambiguous (Shimizu-Motohashi et al., 2019). With regard to rescuing dystrophin expression, the level of dystrophin required for clinical efficacy has remained unclear; while restoring 15%–20% of normal dystrophin levels in mouse models has shown improvement, 33%–40% has been reported as necessary for improvement in dog models (Sun et al., 2020). In humans, restoring 30% of normal dystrophin levels has been suggested as the standard for notable improvement (Sun et al., 2020). However, the relevance of this is still unknown, as some patients with low levels of dystrophin have been shown to maintain relatively normal muscle function, and studies in DMD patients that have extremely low but still detectable levels of dystrophin have suggested that even a small amount of dystrophin protein can mitigate skeletal muscle deficits (Nakamura et al., 2016; Waldrop et al., 2018).

Leading approaches in novel DMD therapies thus far have been gene-based, including gene replacement, endogenous gene repair, exon skipping, and read-through therapies (Yao et al., 2021). Multiple issues with these techniques have arisen, including immune response to the vector, difficulty delivering genes to post-mitotic muscle fibers, and the fact that many of these techniques only have potential for treating DMD patients with particular genetic mutations, of which there are several (Cossu and Sampaolesi, 2007; Iftikhar et al., 2021). Other methods being explored include upregulation of utrophin, inhibition of histone deacetylase, antagonization of the myostatin pathway, and increased angiogenesis (Cossu and Sampaolesi, 2007; Verma et al., 2018; Sun et al., 2020). Agents have also been explored in order to target downstream pathological changes seen in DMD, such as fibrosis, inflammation, ischemia, oxidative stress, loss of calcium homeostasis, and muscle atrophy (Yao et al., 2021). Though exon-skipping and read-through therapies in particular have shown promise, they have their drawbacks and other approaches have shown limited success in preclinical and clinical trials thus far; therefore, there is a distinct need for further research and development.

Of note, several model organisms have been used to study DMD and potential therapies, the most common of which is the *mdx* mouse. The *mdx* mouse has a mutation in the dystrophin gene itself, similar to human DMD patients (Yucel et al., 2018). However, *mdx* mice do not exhibit a shortened lifespan, severe muscle degeneration, or other key features of DMD, such as cardiomyopathy (Yucel et al., 2018; Gao et al., 2019). Their milder phenotype is likely attributable to the high regenerative capacity of mouse muscle, as well as the fact that utrophin is still active, whose expression may compensate for the lack of dystrophin (Plantié et al., 2015; Gao et al., 2019). The utrophin protein has a remarkably similar structure and function to dystrophin in linking the sarcolemma to the cytoskeleton, but is primarily expressed in skeletal muscle during fetal development and, in healthy muscle, is downregulated prior to birth (Yucel et al., 2018). Utrophin is usually upregulated in skeletal muscle when dystrophin is absent and has even been proposed as an alternative target protein to dystrophin, due to their similarities (Yao et al., 2021). However, due to key functional differences in the protein structure, utrophin cannot fully compensate for the loss of dystrophin in human DMD patients (Yucel et al., 2018). In order to account for this in murine models, double knockout (dKO) mice models have been utilized, which, in addition to knocking out *dystrophin*, also knock out one of a variety of genes that play important roles in myogenesis and muscle function (Yucel et al., 2018). Namely, *dystrophi*n/*utrophin* dKO mice have been employed in studies, as they more closely resemble the clinical manifestations of DMD and have been deemed more suitable for gene therapy testing (Gao et al., 2019). Furthermore, a golden retriever muscular dystrophy (GRMD) dog model has come into use, as it better aligns with the progressive course of human DMD than most mouse models (Kornegay, 2017). GRMD studies have not always substantiated findings in *mdx* mouse studies, and have also illuminated various side effects of potential treatments that are not seen in *mdx* mice (Kornegay, 2017). GRMD has also been crucial in investigating the speed of disease progression, as RNA sequencing has been employed to identify biomarkers for this (Brinkmeyer-Langford et al., 2018). Despite their utility, practical considerations do limit the use of GRMD models, especially dog availability and expense (Kornegay, 2017). Finally, *drosophila* and zebrafish models of DMD have been studied, as these organisms have modeled many human diseases over the last several decades and comparative genomic studies have demonstrated sequence conservation of dystrophin in both organisms (Plantié et al., 2015). In particular, zebrafish *sapje* mutants have been useful, as they contain a mutation at *dystrophin* and do not express compensatory *utrophin*, so they exhibit features more similar to human DMD pathology (Plantié et al., 2015).

## Skeletal muscle regeneration

Because muscular dystrophies, including DMD, are characterized by progressive degeneration of skeletal muscle, the structure and function of skeletal muscle must be understood in order to develop effective therapeutic strategies. In humans, skeletal muscle comprises approximately 40% of total body weight (Frontera and Ochala, 2015). It is responsible for converting chemical energy into mechanical energy, hence generating force and power to produce movement and perform voluntary functions (Frontera and Ochala, 2015). Skeletal muscle is necessary for the maintenance of homeostasis and other bodily functions as well, including respiration and metabolic function (Joanisse et al., 2017; Sousa-Victor et al., 2022).

Skeletal muscle is heterogeneous tissue, composed of different types of muscle fibers (Joanisse et al., 2017). Its architecture involves a specific arrangement of these muscle fibers, along with surrounding connective tissue (Frontera and Ochala, 2015). Skeletal muscle fibers are formed through the fusion of single cells and contain several hundred post-mitotic nuclei in their mature form (Tedesco et al., 2010). This multinucleation presents a hurdle to the treatment of deficient skeletal muscle, as therapies face the task of restoring proper gene expression in hundreds of millions of post-mitotic nuclei (Tedesco et al., 2010).

Skeletal muscle tissue has the ability to regenerate new muscle fibers upon indication by homeostatic demand, hypertrophy, or need for repair secondary to injury, exercise, or disease (Tedesco et al., 2010; Sato et al., 2012). The regenerative capacity of skeletal muscle declines with aging (Sousa-Victor et al., 2022). The stem cells of postnatal muscle, known as satellite cells, are responsible for >99% of the regenerative potential of adult skeletal muscle (Conboy et al., 2003; Bröhl et al., 2012; Joanisse et al., 2017). Other progenitors, including pericytes, endothelial cells, and interstitial cells, have a limited amount of regenerative potential as well (Tedesco et al., 2010; Verma et al., 2018).

## Satellite cells

Satellite cells are crucial to the regenerative capacity of skeletal muscle tissue; when they dysfunction, the skeletal muscle loses its regenerative ability, leading to the degeneration reminiscent of DMD (Asakura et al., 2002; Bröhl et al., 2012; Sato et al., 2012; Fuchs and Blau, 2020). During prenatal development, some muscle progenitor cells migrate into position as mononuclear cells between the basal lamina of the muscle fiber and its sarcolemma; these cells ultimately constitute the satellite cell pool and are defined by their unique molecular location (Tedesco et al., 2010; Wilschut et al., 2012). Satellite cells are mononuclear and comprise 2.5%–6% of all nuclei for any given skeletal muscle fiber (Tedesco et al., 2010). They play important roles both in establishing and growing muscle during development and in maintaining muscle in adults via regeneration (Bröhl et al., 2012). Satellite cells are distinct from other types of progenitors in that they are limited to myogenesis, rather than possessing a broader multilineage differentiation potential (Asakura et al., 2001; 2002; Wilschut et al., 2012).

Satellite cells can adopt several different states, including quiescence, activation, proliferation, and differentiation (Li et al., 2021). Initially, during the perinatal and postnatal periods, satellite cells are proliferative (Bröhl et al., 2012). Then, in healthy adult muscle, satellite cells maintain the essential feature of quiescence under homeostatic conditions (Bröhl et al., 2012). Quiescence is common to stem cells and is defined by reversible mitotic arrest: quiescent cells do not divide, but rather are still able to re-enter the cell cycle and proliferate upon stimulation (Bjornson et al., 2011). Quiescence permits self-renewal, allowing for the maintenance of stem cell populations throughout the lifetime of an animal (Bjornson et al., 2011). Furthermore, quiescence involves reduced metabolic activity, so that satellite cells are protected against endogenous stress from DNA replication and cellular respiration (Bjornson et al., 2011). On a genetic level, quiescent cells highly express the paired box 7 (*Pax7*) gene, which has been shown to be the most reliable marker for satellite cells (Mourikis et al., 2012; Fuchs and Blau, 2020; Starosta and Konieczny, 2021; Sousa-Victor et al., 2022). Mice null for *Pax7* are notably deficient in satellite cells, and misregulation of *Pax7* has been implicated in DMD (Gayraud-Morel et al., 2012; Coppens et al., 2021). Aside from satellite cells, *Pax7* is not expressed by any other cell type in muscle tissue (Fuchs and Blau, 2020).

Upon injury or due to homeostatic demand, satellite cells leave their quiescent state, re-entering the cell cycle and downregulating *Pax7* (Dumont et al., 2015; Kann et al., 2021). At this point, they instead express myogenic regulatory factors (MRFs), including *Myf5* and *MyoD,* which promote cell activation and the cells become myoblasts, poised to regenerate muscle tissue (Mourikis et al., 2012; Wilschut et al., 2012; Sousa-Victor et al., 2022). MRFs are specifically expressed in skeletal muscle lineage, and when transfected into certain other cell types, MRFs can induce them to adopt a myogenic fate (Biressi et al., 2013). Cell ablation studies have demonstrated that *Myf5* and *MyoD* can independently initiate myogenic differentiation, thus deeming them myogenic determination genes (Biressi et al., 2013). *Myf5* is the first MRF to be expressed during mammalian development (Biressi et al., 2013). Though *MyoD* is only expressed in a small percentage of quiescent satellite cells, all progenitors of satellite cells transcribe *MyoD* prenatally and express it prior to the first cell division (Kanisicak et al., 2009; Gayraud-Morel et al., 2012). Other MRFs include *Mrf4*, which only acts as a determination gene during embryonic development, and *myogenin*, which is never co-expressed with *Pax7* and acts as a differentiation factor downstream of *MyoD* and *Myf5* (Kottlors and Kirschner, 2010; Gayraud-Morel et al., 2012; Biressi et al., 2013).

Importantly, the satellite cell population is heterogenous, and subpopulations serve distinct functions (Dumont et al., 2015). Some primarily perform asymmetric divisions, which are more prevalent during homeostasis in order to generate myogenic progenitors while maintaining the satellite cell pool (Dumont et al., 2015; Sousa-Victor et al., 2022). Others predominantly perform symmetric divisions, which are more common after indication by injury or breakdown, as they act to expand the satellite cell pool (Dumont et al., 2015; Sousa-Victor et al., 2022). Satellite cells maintain a dynamic balance between symmetric and asymmetric division in accordance with the present needs of the muscle, and which type of division that satellite cells undergo is primarily governed by two factors: polarity and orientation (Dumont et al., 2015). Asymmetric division is driven by unequal distribution of polarity proteins, specifically members of the Partitioning-defective protein (PAR) family, which establish cell polarity in several different cell types (Dumont et al., 2015). With regard to orientation, symmetric divisions typically occur in a planar orientation, while asymmetric divisions usually occur in an apical-basal orientation (Kuang et al., 2007). Furthermore, different subpopulations of satellite cells express genes associated with quiescence and myogenesis to varying degrees (Dumont et al., 2015). Though *MyoD* is consistently expressed in all activated satellite cells, there are populations of *Myf5-*positive and *Myf5-*negative satellite cells present in adult muscle; studies using fluorescent protein tagging have revealed that approximately 10% of satellite cells constitute a subpopulation that never expresses *Myf5* during development (Kuang et al., 2007; Dumont et al., 2015; Sousa-Victor et al., 2022). Interestingly, these *Myf5-*negative cells have shown a greater ability to self-renew and are more stem-like as compared to their *Myf5-*positive counterparts (Kuang et al., 2007; Sousa-Victor et al., 2022). Also, satellite cells demonstrate *Pax7* at different levels, and subpopulations of satellite cells that express higher levels of *Pax7* are less prone to differentiation (Rocheteau et al., 2012). This is not surprising since *Pax7* is so intimately associated with quiescence and proliferation.

The intrinsic regulatory mechanisms that govern quiescence, cell cycle progression, and cell fate determination of satellite cells are influenced by extrinsic cues (Mourikis et al., 2012; Dumont et al., 2015; Verma et al., 2018). These cues involve proximity to endothelial cells, as well as the specialized local niche between the myofiber sarcolemma and basal lamina in which satellite cells reside (Dumont et al., 2015; Verma et al., 2018). During quiescence, the basal lamina physically separates satellite cells from other local niche cells (Kann et al., 2021). Muscular injury induces changes in this niche, namely disruption of the basal lamina, which exposes satellite cells to the growth factors and signaling molecules that they are normally sequestered from (Kann et al., 2021). This external cue subsequently promotes satellite cell activation and proliferation.

## Satellite cells as a therapeutic tool for DMD

Though it has been established that satellite cells drive muscle regeneration and that impaired regeneration drives the phenotype of DMD, the specific role of satellite cells in this impaired regeneration has been disputed (Dumont and Rudnicki, 2016).

Some studies have reported that satellite cell number remains the same or is even elevated in dystrophic muscle as compared with age-matched controls (Kottlors and Kirschner, 2010; Jiang et al., 2014). Further, some studies have suggested that impaired regeneration occurs secondary to the surrounding pathogenic environment rather than intrinsic issues with the regenerative capacity of satellite cells (Boldrin et al., 2015). This would indicate that lack of satellite cells is not the culprit for impaired regeneration. Similarly, other studies have suggested that insufficient regeneration may be caused by failure of differentiation rather than a lack of self-renewal and proliferation (Kottlors and Kirschner, 2010). This presents a direct link between dystrophin deficiency and impaired regeneration, as dystrophin is crucial for defining cell polarity, which strongly influences whether satellite cell division is asymmetric or symmetric and subsequently whether differentiation and myogenesis occur (Chang et al., 2016; Dumont and Rudnicki, 2016). Dystrophin influences polarity by interacting with MARK2, a cell polarity-regulating kinase (Chang et al., 2016). When dystrophin is deficient in DMD muscle, there is reduced expression of MARK2, which leads to loss of PARD3 polarization, a member of the PAR family (Chang et al., 2016). Consequently, asymmetric satellite cell divisions wither, so fewer myogenic progenitors are produced and muscle regeneration is impaired (Chang et al., 2016; Dumont and Rudnicki, 2016). This link between dystrophin and polarity demonstrates a direct role for dystrophin in the loss of regenerative capacity seen in DMD, and supports the idea that exhaustion of the satellite pool may not be the cause for impaired regeneration.

On the contrary, several studies have indicated that satellite cell pool exhaustion precipitates loss of regenerative capacity, and satellite cells do remain a key target for therapies combatting DMD (Wilschut et al., 2012). Due to their constant activation in an attempt to compensate for the unrelenting muscle degeneration of DMD, the satellite cell pool becomes depleted in dystrophic tissue, leading to failure of muscle repair and accelerated disease progression (Morgan and Zammit, 2010; Sacco et al., 2010; Wilschut et al., 2012; Jiang et al., 2014; Mu et al., 2015). Hence, rescuing satellite cell functionality has been a key target for DMD therapies (Liu et al., 2021).

The year 1990 saw the first successful satellite cell transplant into a boy with DMD, which achieved dystrophin production (Law et al., 1990). Though clinical trials throughout the 1990s demonstrated safety, no significant functional benefit was identified (Cossu and Sampaolesi, 2007). Approaches to satellite cell transplantation are still being explored in order to account for their drawbacks. For example, the heterogeneity of cultured satellite cells makes them somewhat unreliable in transfer therapies, so they have been cultured in various systems or on different mediums in order to select satellite cells that can best survive, engraft, and repopulate (Sun et al., 2020). Due to the lack of adequate success with satellite cell transfer thus far, the quest for optimization of this approach persists.

Though satellite cells have been looked to as a superior option, as they have self-renewal capacity and differentiation potential, myoblast-transfer therapies have been explored as well, as they have a high ability to generate muscle fibers (Shimizu-Motohashi et al., 2019). Pioneer myoblast-transfer studies demonstrated that intramuscular injection of healthy myoblasts into *mdx* mice resulted in fusion with host fibers and extensive dystrophin production (Tedesco et al., 2010). These early suggestions of success led to several clinical trials in the early 1990s–2000s that unfortunately failed for various reasons, including poor survival, limited migration of donor cells after transplantation, and immune responses that caused the rejection of the donor cells (Tedesco et al., 2010; Shimizu-Motohashi et al., 2019). In the interim, numerous approaches with different transplantation techniques have been attempted to sidestep these concerns, including employing conditional proliferation-dependent suicide agents in order to combat the oncogenic potential of immortalized cells (Sun et al., 2020). However, ample success has not yet been achieved with myoblasts (Sakai et al., 2017).

In addition to satellite cells and myoblasts, several different myogenic progenitors have been investigated as potential candidates in transfer therapies as well, including mesenchymal stem cells, CD133^+^ cells, mesangioblasts, embryonic stem cells, and induced pluripotent stem cells (Sakai et al., 2017; Shimizu-Motohashi et al., 2019; Tominari and Aoki, 2022). Despite the established efficacy of exon-skipping and read-through therapies, cell transplantation therapies are applicable to more patients, as they are not specific to patients with certain types of mutations (Tominari and Aoki, 2022). Cell transfer therapies have both explored using genetically unmodified cells from healthy donors, which are mutation-free but carry a greater risk of immune reaction, and using autologous genetically-corrected cells, which require gene manipulation prior to transplantation but carry a much lower risk of immune reaction (Shimizu-Motohashi et al., 2019; Duan et al., 2021). Clinical trials in human patients thus far have primarily focused on cells from healthy donors, while preclinical trials in animals have employed the autologous genetically-corrected cell approach (Shimizu-Motohashi et al., 2019). As discussed with regards to satellite cell- and myoblast-transfer therapies, promise for these approaches has been demonstrated but they have not been overwhelmingly successful thus far.

## Notch signaling system

Satellite cells are an important target to consider in novel therapies since their depletion is a strong potential cause for DMD progression. Therefore, it is essential that we deepen our understanding of the various pathways that regulate their function. One of these regulators is the Notch signaling system, whose dysfunction has been shown to specifically deplete satellite cells, as is seen in DMD (Jiang et al., 2014). The Notch signaling pathway is highly conserved in both vertebrates and invertebrates and regulates cell proliferation, cell fate decisions, and induction of differentiation (Bröhl et al., 2012; Sato et al., 2012; Starosta and Konieczny, 2021). The Notch pathway serves important roles in both embryonic development and in adults (Luo et al., 2005). During development, Notch signaling is crucial for formation of healthy muscle, as elimination of the pathway has been shown to lead to premature differentiation of myogenic progenitors and ultimately to the development of small and weak muscle groups (Bröhl et al., 2012; Mourikis et al., 2012). Furthermore, embryonic knockouts of various components of the Notch pathway genes have been shown to be lethal in mice, deeming it essential for embryonic development (McIntyre et al., 2020). In adults, Notch signaling is crucial for tissue renewal and maintenance in multiple organ systems, with one of its primary roles being regulation of myogenesis and the regeneration of skeletal muscle tissue (Conboy and Rando, 2002; Sato et al., 2012; Coppens et al., 2021). With old age, Notch signaling decreases in skeletal muscle, leading to an age-related decline in the proliferative ability of satellite cells and subsequently to lower regenerative potential (Conboy et al., 2003). The pathway is context-dependent, altering based on factors such as cell type, timing, and mode of signaling, so that it can respond to the current needs of the organism (Kuroda et al., 1999).

**FIGURE 1:**
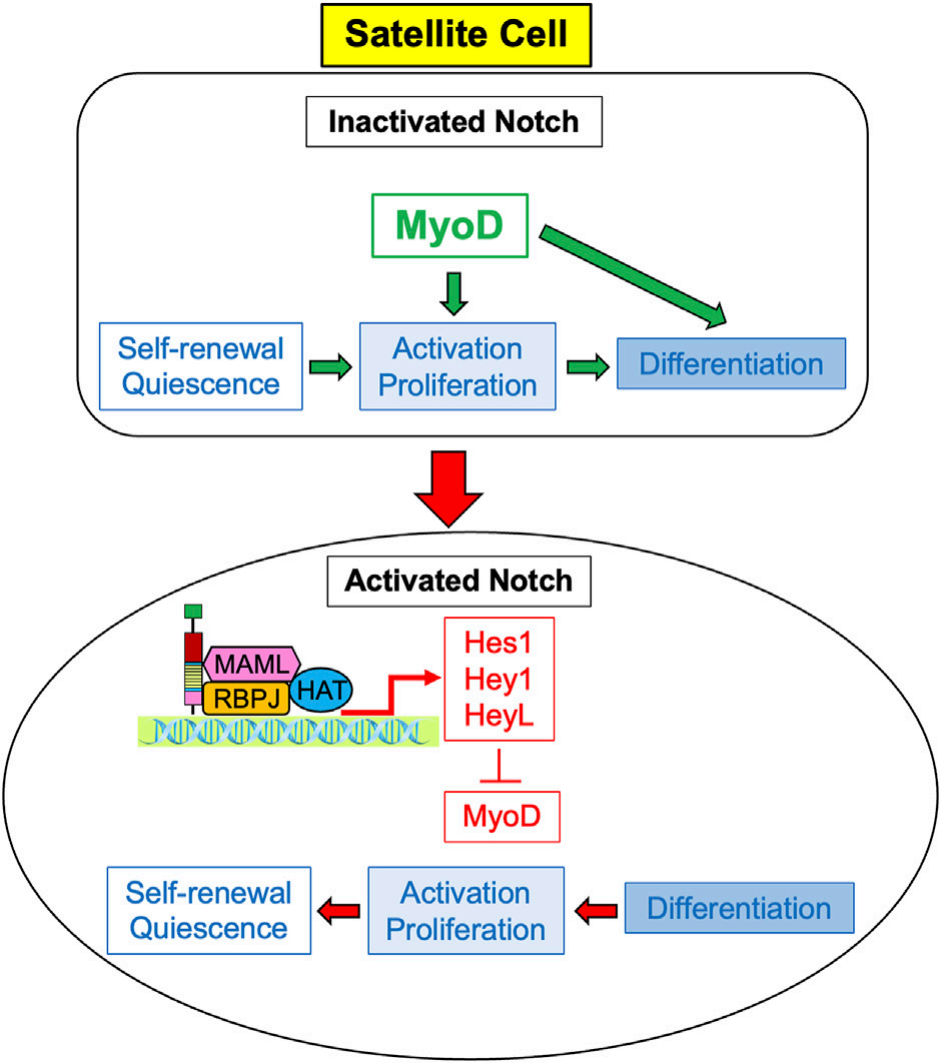
Notch signaling induces proliferation of satellite cells. Expression of MyoD causes activation, cell the proliferation and differentiation of satellite cells, so they ultimately cease cell division and differentiate into myocytes. Activated Notch signaling results in the expression of Notch target genes *Hes1*, *Hey 1*, and *HeyL*, which inhibit the transcription of MyoD. Without MyoD, satellite cells predominately proliferate rather than differentiate, and ultimately self-renew to return to quiescent cells.

In order to promote the regeneration of skeletal muscle in the adult, Notch signaling regulates satellite cells by suppressing myogenic differentiation (Mourikis et al., 2012). When Notch is inactivated, MyoD activates satellite cells causing their differentiation. However, activated Notch promotes the expression of Hes and Hey family genes, particularly *Hes1, Hey1,* and *HeyL* (Sakai et al., 2017; D’Souza et al., 2008). These genes suppress MyoD, and therefore suppress differentiation while promoting satellite cell proliferation and self-renewal in order to maintain the satellite cell pool, as depicted in Figure 1 (Gioftsidi et al., 2022). Maintenance of the satellite cell pool then augments the regenerative ability of the skeletal muscle. Unsurprisingly, active Notch signaling is associated with the maintenance of quiescence; in quiescent satellite cells, Notch signaling is high and Notch target genes are more highly expressed to promote self-renewal and proliferation, whilst MyoD and differentiation are suppressed (Fukada et al., 2007; Bjornson et al., 2011; Baghdadi et al., 2018; Kann et al., 2021; Sousa-Victor et al., 2022). Several studies in both *mdx* mice and in satellite cell-derived myoblasts have also demonstrated that constitutive Notch activation upregulates *Pax7*, which is associated with satellite cell quiescence and self-renewal (Wen et al., 2012; Jiang et al., 2014).

It should be noted that Notch signaling plays a role in muscle cells beyond just satellite cells, as well. Importantly, Notch1 may be activated in myotubes, which serves to upregulate the expression of Notch ligands and enhance the regenerative capacity of adjacent satellite cells (Bi et al., 2016). This aspect enhances the ability of myotubes as a stem cell niche. Furthermore, it has been shown that in disuse and diabetes, multinucleated myofibers express Notch2 via activation by the Notch ligand Dll4 from the microvascular endothelium (Fujimaki et al., 2022). This ultimately leads to the progression of muscle atrophy seen in these conditions, making it a therapeutic target (Fujimaki et al., 2022).

## Notch signaling scheme

**FIGURE 2:**
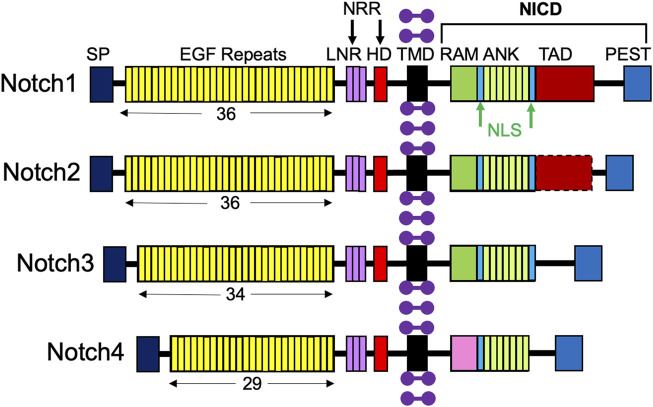
Structures of the four Notch receptors. The four Notch receptors identified in humans are depicted with their structural components: SP, signal peptide; EGF, repeats indicated by the number below; LNR, LIN12-Notch repeats; HD, heterodimerization domain; NRR, negative regulatory region; TMD, transmembrane domain; RAM, RBPJ-associated module; NLS, nuclear localization signal; ANK, ankyrin repeats; TAD, transcriptional activation domain; PEST (proline/glutamic acid/serine threonine-rich motif), and NICD, Notch intracellular domain. Purple dumbbells represent surrounding membrane phospholipids.

A molecular scheme for the Notch signaling pathway has been established, lending insight into ways in which it can be targeted in therapies. Notch receptors are large type I transmembrane proteins comprised of an extracellular domain, a single transmembrane domain, and an intracellular region (Figure 2; Sato et al., 2012). There are four different Notch receptors in humans, Notch1-4, which are structurally distinct. Their extracellular domain includes a signal peptide, 29–36 epidermal growth factor (EGF) repeats, three conserved cysteine-rich Lin12-Notch repeats (LNR), and a heterodimerization domain (HD); together, the LNR and HD constitute the negative regulatory region (NRR), which keeps the receptor “off” when there are no ligands present (Sato et al., 2012). The Notch intracellular domain (NICD) consists of a recombining binding protein - J (RBPJ) association module domain (RAM), nuclear localization signals (NLS), seven ankyrin repeats (ANK), a transcriptional activation domain (TAD), and a C-terminal proline-glutamic acid/serine threonine-rich motif (PEST) (Sato et al., 2012). Importantly, Notch3 and Notch4 lack a TAD (Fujimaki et al., 2017).

**FIGURE 3:**
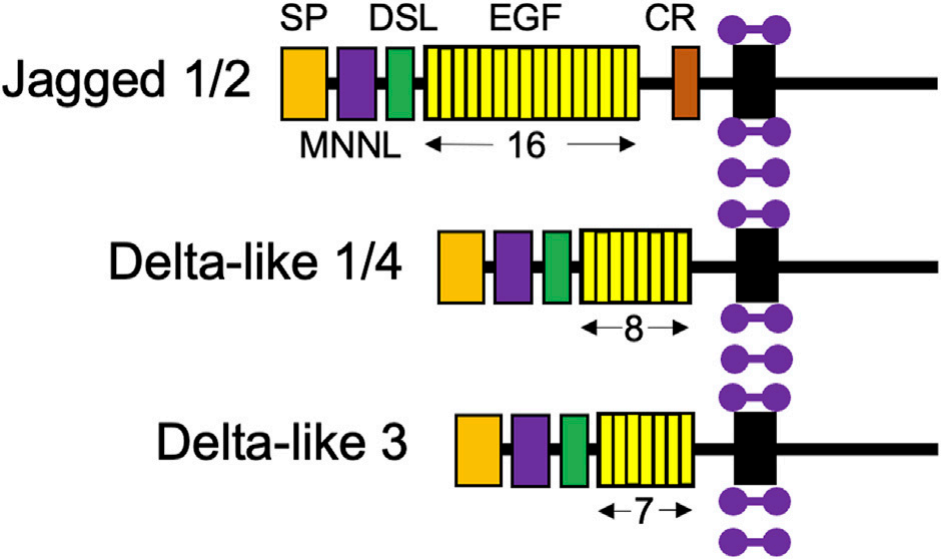
Structures of the five Notch ligand proteins. The five Notch ligands identified in humans are depicted with their structural components: SP, signal peptide; MNNL, modular N-terminal Notch ligand; DSL, Delta, Serrate, and LAG-2 domains; EGF, repeats indicated by number below; and CR, cysteine-rich domain. Purple dumbbells represent surrounding membrane phospholipids.

Like Notch receptors, Notch ligands are also type 1 cell surface proteins with multiple tandem EGF repeats in their extracellular domains (Figure 3; D’Souza et al., 2008). There are five Notch ligands in humans, Jagged1, 2 (Jag1, 2) and Delta-like 1, 3, and 4 (Dll1, 3, 4), which each have unique structures. Notch ligands are presented on neighboring niche cells and activate Notch receptors through a juxtracrine pathway (Coppens et al., 2021; Kann et al., 2021). Of note, there has been evidence to demonstrate *cis*-inhibitory interactions between Notch ligands and receptors located on the same cell (Coppens et al., 2021).

**FIGURE 4:**
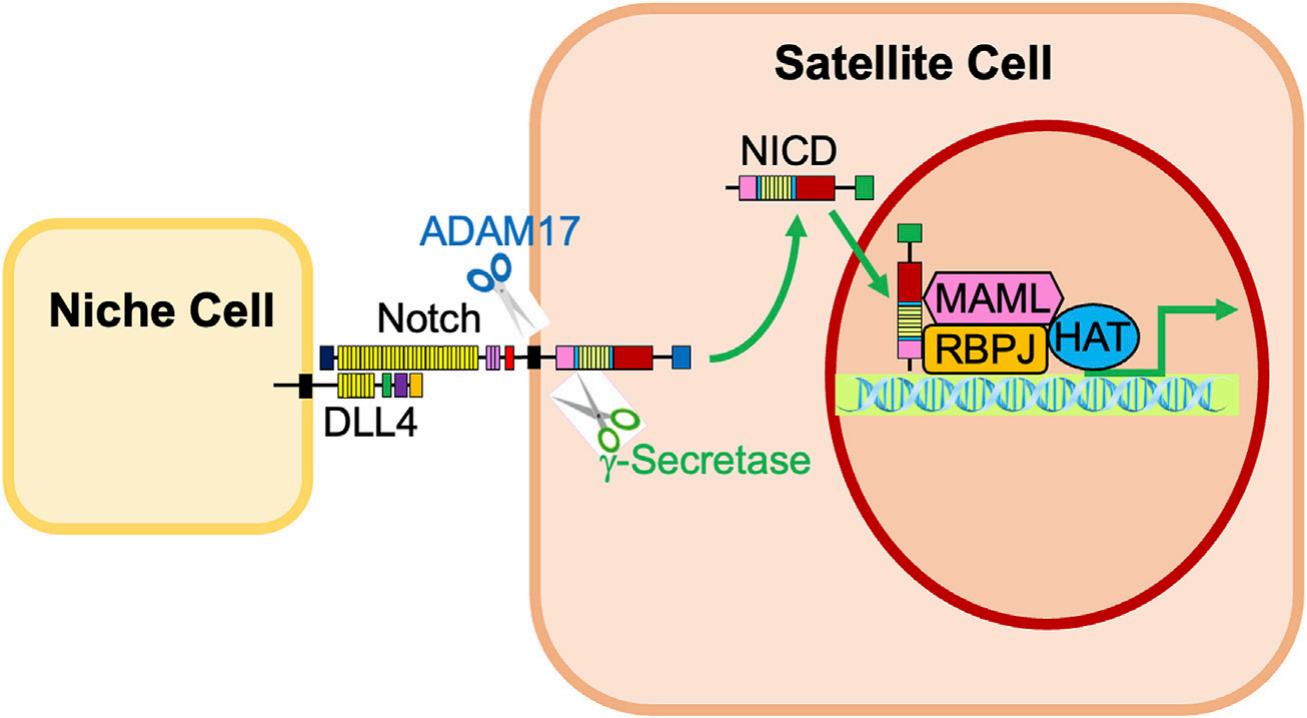
Activation of Notch signaling in satellite cells by neighboring niche cells. Notch receptors expressed in satellite cells are activated by interaction with Notch ligands, such as Dll4, on neighboring niche cells. The Notch receptor is cleaved by ADAM17 and γ-secretase, liberating the Notch Intracellular Domain (NICD). The NICD translocates into the nucleus, associates with RBPJ, and recruits transcriptional coactivators, including Mastermind (MAML) and histone acetyltransferases (HAT). This complex promotes the activation of Notch target genes *Hes1, Hey1,* and *HeyL*.

After the ligand binds to the extracellular domain of the Notch receptor, a signal transduction cascade is initiated, known as the canonical Notch signaling pathway, depicted in Figure 4 (McIntyre et al., 2020). Initially, the receptor undergoes a series of proteolytic cleavages, first by an ADAM-family metalloproteinase in the extracellular domain and then by γ-secretase in the transmembrane region, in order to ultimately liberate the Notch intracellular domain (NICD) (McIntyre et al., 2020). This step presents an opportunity for regulation of the Notch pathway; if cells are treated with DAPT, γ-secretase is inhibited, NICD will therefore not be liberated, and transcription of target genes will not be activated (Dong et al., 2021). Once liberated under normal conditions, NICD translocates to the nucleus and associates with a DNA-binding protein that includes RBPJ (Nandagopal et al., 2018; Kann et al., 2021). Of note, RBPJ is also known as the CSL (CBF1/Suppressor of Hairless/LAG-1) (McIntyre et al., 2020). Without NICD, RBPJ binds transcriptional corepressors to inhibit the transcription of target genes (Bjornson et al., 2011). When NICD is present, it binds RBPJ, displaces the corepressors, and recruits coactivators, including Mastermind (Mastermind-like in mammals) and histone acetyltransferases to assemble a transcriptional complex and activate transcription of Notch target genes, including *Hes1, Hey1,* and *HeyL* (Figure 1, 2; McIntyre et al., 2020; D’Souza et al., 2008; Bröhl et al., 2012; Sato et al., 2012; Baghdadi et al., 2018). Intracellular Notch activity is regulated by protein turnover that occurs at the PEST at the C-terminal domain of the NICD, as the NICD rapidly degrades when targeted by ubiquitylation (Andersson et al., 2011).

## Various notch receptors and ligands

Multiple studies have demonstrated that the Notch receptors and ligands are not functionally equivalent, having varied downstream effects on self-renewal, proliferation, and differentiation of satellite cells (Preuße et al., 2015; Nandagopal et al., 2018). These differences are important to understand, especially when considering how to optimally target Notch in novel therapies.

With regards to receptors, prior studies have demonstrated that Notch1 and Notch3 have different and even contradictory roles, with Notch3 acting as a repressor for Notch1 (Low et al., 2018). Whereas activity of the canonical Notch signaling pathway typically leads to an increase in proliferation of satellite cells, experiments have shown that cells only expressing Notch3 proliferate less than controls and, similarly, that deficiency of Notch3 even leads to increased muscle regeneration and higher numbers of satellite cells (Kitamoto and Hanaoka, 2010). Additionally, Fujimaki et al. demonstrated that knockout of either Notch1 or Notch2 in satellite cells in mice prevents proliferation and self-renewal, indicating that they are necessary for the maintenance of the satellite cell pool and adult muscle regeneration (Fujimaki et al., 2017). On the contrary, mice deficient in Notch3 were viable and fertile, and even exhibited elevated numbers of quiescent satellite cells and greater proliferative ability, again suggesting that Notch3 acts to negatively regulate satellite cell proliferation (Fujimaki et al., 2017).

Numerous studies have specifically evaluated the ability of ligands Dll1, Dll4, Jag1, and Jag2 to induce Notch signaling, with results proving to be somewhat contradictory (Sakai et al., 2017). In human cells, *in vitro* studies have shown that Dll4 and Jag1 more strongly induce Notch signaling than does Dll1 (Sakai et al., 2017). Moreover, differences between Dll1 and Dll4 have been identified with respect to both the manner in which they activate Notch1 and their downstream effects, and it has been established that they are unable to replace the function of one another in many tissues (Preuße et al., 2015). First, Dll4 is presented to satellite cells by adjacent myofibers during quiescence, whereas Dll1 is expressed by differentiating cells that provide a self-renewing signal during regeneration (Yartseva et al., 2020; Kann et al., 2021). Second, Dll1 and Dll4 activate Notch1 in distinct manners: Dll1 activates Notch1 in discrete, frequency-modulated pulses, while Dll4 activates Notch1 in a sustained, amplitude-modulated manner (Nandagopal et al., 2018). Satellite cells are able to discriminate between these two types of signals using dynamics, with the Dll1 signal primarily upregulating *Hes1* and the Dll4 signal primarily upregulating *Hey1* and *HeyL* (Nandagopal et al., 2018; Zhang et al., 2021). Different ligands also affect satellite cell activity differently in distinct environments as a stem cell niche. Recent work has demonstrated that proximity to blood vessels is associated with satellite cell self-renewal as a vascular niche, specifically when Dll4 induces quiescence, therefore creating a vascular niche for satellite cell maintenance (Verma et al., 2018).

## Notch signaling in DMD

The absence of Notch signaling precipitates impaired muscle regeneration and phenotypic characteristics reminiscent of DMD (Siebel and Lendahl, 2017). Lin et al. showed that conditional knockout mice whose endogenous Notch signaling was blocked in the satellite cell compartment acquired several features of muscular dystrophy, namely impaired muscle regeneration (Lin et al., 2013). Furthermore, inhibition of Notch signaling in satellite cells led to reduced self-renewal capacity and proliferation as well as increased differentiation of myoblasts, which together exhaust the satellite cell pool (Lin et al., 2013; Verma et al., 2018; Fiore et al., 2020). Together, these results suggest that Notch signaling promotes processes necessary for muscle tissue regeneration, and it is thus likely that impaired Notch signaling contributes to the pathogenic mechanisms of DMD (Tables 1, 2). On that note, insufficient Notch signaling has been directly implicated in DMD, as recent works demonstrated that DMD muscle tissue contains decreased Notch signaling (Table 1; Church et al., 2014; Starosta and Konieczny, 2021), although an earlier work showed upregulation of Notch signaling genes in *mdx* mice (Table 1; Turk et al., 2005). Jiang et al. showed that satellite cells in the skeletal muscle of *mdx* mice exhibit reduced expression of multiple Notch receptors, ligands, and target genes such as *Hes1*, *Hey1* and *HeyL* (Table 1; Jiang et al., 2014). Matrix metalloproteinase-9 (MMP9) is an extracellular protease involved in tissue remodeling, inflammation, and development of interstitials in skeletal muscle (Hindi et al., 2013). Gene knockout of *Mmp9* increases the expression of Notch ligands and receptors, and Notch target genes in skeletal muscle of *mdx* mice, and dramatically improves myopathy and augments myofiber regeneration in *mdx* mice (Table 1; Hindi et al., 2013).

**TABLE 1:** Aberrant expression of Notch signaling genes in muscular dystropjy.

Gene	Species	Function	Citation
DII3, Numb, Numbl, Notch1, Notch2	Mouse	Upregulated Notch signaling genes in mdx mice	Turk et al. (2005)
Notch2, Notch3, Jag2, Hes1, HeyL	Mouse	Upregulated Notch signaling genes inmdx:Mmp9^+/−^mouse muscle compared with mdx mice	Hindi et al. (2013)
Jag2, Numb	Mouse	Both genes are downregulated in mdx mice	Church et al. (2014)
Jag2, Notch1, Notch2, Numb, Hes1	Mouse	These genes are downregulated in mdx:utrn−/− dKO mice	Church et al. (2014)
Numb, Notch3	Human	Both genes are upregulated in DMD.	Church et al. (2014)
Notch1, Hes1	Human	Both genes are downregulated in DMD.	Church et al. (2014)
Notch1, Notch3, Jag1, Hey1, HeyL	Mouse	Notch1, Notch3, Jag1, Hey1 and HeyL are reduced in the mdx myoblasts	Jiang et al. (2014)
Notch1, Notch2, Notch3, Jag2, Hes, Hey1	Mouse	Downregulation of these genes in the skeletal muscles of mdx, mice	Mu et al. (2015)
Notch1, Notch2, Notch3, Jag2, Hes1, Hey1	Mouse	Over-activation of these genes in the skeletal muscles of mdx:utrn−/− dKO mice	Mu et al. (2015)
Notch1	Human	POGLUT1-mutated muscular dystrophy patients revealed decrease in the level of the NOTCH1	Servián-Morilla et al. (2016)
Jag1, DII1	Mouse	Expression of DII1 and Jag1 is higher in mdx:PKCθ−/− mouse muscle compared with mdx mice	Fiore et al. (2020)

**TABLE 2:** Phenotypical changes of muscular dystrophy via modulation of Notch signaling.

Gene	Species	Function	Citation
MEGF10	Human	Early-onset myopathy, areflexia, respiratory distress and dysphagia (EMARDD) or MEGF10 myopathy is associated with mutations in MEGF10, in which satellite cells from the patients show impaired proliferation and differentiation	Logan et al. (2011)
Notch	Mouse	mdx satellite cells have reduced activation of Notch signaling	Jiang et al. (2014)
Notch1	Mouse	Notch1 activation is sufficient to rescue the self-renewal deficiencies of mdx satellite cells	Jiang et al. (2014)
Notch	Mouse	Notch inhibition produces functional defects in mdx muscle	Church et al. (2014)
Jag1	Dog	Overexpression of Jag1 rescues the DMD Phenotype in golden retriver muscular dystrophy (GRMD)	Vieira et a., 2015
Notch	Mouse	Inhibition of Notch by treatment with DAPT improves DMD phenotypes in mdx:utrn−/− dKO mice	Mu et al. (2015)
POGLUT1	Human	A POGLU1 mutation causes lim-girdle muscular dystrophy (LGMD) with reduced Notch	Servián-Morilla et al. (2016)
Notch1	Mouse	Myofiber-specific activiation of Notch1 improves muscle pathology in mdx mice	Bi et al. (2016)
Jag1	Human	Jag1 induced IL-1β in DMD but not by normal myogenic cells reduces proliferation and differentiation	Nagata et al. (2017)
MEGF10	Mouse	Satellite cells from Meg10−/− mice and Megf10−/−:mdx dKO mice also show impaired proliferation and migration	Saha et al. (2017)
Notch2NLC	Human	CGG expression in NOTCH2NLC is associated with oculopharyngodistal myopathy (OPDM)	Ogasawara et al. (2020)
Notch/TGFb	Human	Inhibition of Notch and TGFβ promotes mygenic differentiation of human DMD iPSCs	Choi et al. (2012)
Jag 2	Human	Jag2 mutations are associated with unsolved muscular dystrophy	Coppens et al. (2021)

Fiore et al. demonstrated that loss of or pharmacological inhibition of protein kinase C-θ (PKCθ), which modulates various signaling pathways in muscle, led to increased Notch signaling in *mdx* mice, and consequently improved muscle regeneration and reduced muscle wasting. This study also noted that inhibition of PKCθ acted to upregulate the expression level of *Pax7* and *Notch1*; together, these results demonstrate an avenue for increasing Notch pathway activity to ameliorate the DMD phenotype (Table 1; Fiore et al., 2020).

Specific components of the Notch pathway, namely ligands Jag1 and Jag2, have been implicated in the dysfunction seen in muscular dystrophy. One large study identified the over-representation of pathogenic *Jag2* variants in patients with genetically-unsolved muscular dystrophy (Table 2; Coppens et al., 2021). Also, the downregulation of *Jag2* in murine myoblasts has been connected with the downregulation of other components of the Notch pathway, suggesting its importance (Coppens et al., 2021). *Jag1* has been closely tied to DMD, especially in a GRMD DMD model dog (Table 2; Vieira et al., 2015). Whole genome studies have uncovered an SNP in the promoter region of GRMD *Jag1* that creates a novel myogenin binding site (Vieira et al., 2015; Gioftsidi et al., 2022). This allows for greater expression of *Jag1* via myogenin, a transcription factor in the MyoD family, which leads to greater proliferative potential (Vieira et al., 2015; Gioftsidi et al., 2022). Not only has greater proliferative capacity been observed with overexpression of *Jag1*, but this intervention can even ameliorate the DMD phenotype in GRMD, marking it as a potential therapeutic target and indicating that *Jag1* may act as a genetic modifier of DMD (Vieira et al., 2015; Gioftsidi et al., 2022). Similarly, overexpression of *Jag1* in the *sapje*, a zebrafish model of DMD, produced a fiber organization resembling the wild-type phenotype (Vieira et al., 2015; Gioftsidi et al., 2022).

Despite promising evidence for restoration of Notch signaling or upregulation of components of the Notch pathway to benefit DMD, other reports have been inconsistent. In one study, genes for Notch signaling were downregulated in *mdx* satellite cells and Notch1 activation was sufficient to rescue the self-renewal deficiencies of *mdx* satellite cells, but failed to improve muscle pathology (Tables 1, 2; Jiang et al., 2014). Another study concluded that though Notch inhibition does produce slight functional defects in dystrophic muscle, Notch activation does not significantly improve muscle regeneration in mouse models (Table 2; Church et al., 2014). So, despite some promising experiments, further research is needed to clearly elucidate the link between muscular dystrophy and Notch signaling and set the stage for effective therapies.

To add to these contradictory findings, some studies have found overexpression of Notch signaling to actually be associated with the DMD phenotype, whereas deficiency of Notch signaling is usually thought to precede the satellite cell pool depletion implicated in DMD (Mu et al., 2015). Studies in *dystrophi*n/*utrophin* dKO mice have exhibited sustained inflammation, impaired muscle regeneration, and rapid depletion and senescence of satellite cells associated with overactivation of Notch signaling genes (Table 1; Mu et al., 2015). The reasoning for this is that Notch signaling may repress myogenesis, which causes it to adversely affect muscle regeneration (Mu et al., 2015). Subsequent experiments showed that intramuscular injection of DAPT, a γ-secretase inhibitor, acted to inhibit Notch signaling and consequently upregulated expression of Pax7 and MyoD, and also improved the histopathology of dystrophic muscle (Table 2; Mu et al., 2015). This suggests that activated Notch signaling may participate in the pathology of DMD, and that downregulation of Notch may be an effective therapeutic approach (Mu et al., 2015). Importantly, these experiments utilized dKO mice, rather than the *mdx* mouse model that has been more frequently employed. It has been suggested that findings in dKO mice may more accurately reflect phenomena in human DMD, as these models more accurately mimic DMD pathology (Gao et al., 2019).

Notably, overexpression of Notch exhibits context-dependent effects, depending upon whether it is induced in satellite cells or in differentiated cells (Table 2; Bi et al., 2016). For example, Bi et al. found that overexpression of Notch signaling in satellite cells causes dedifferentiation into quiescent satellite cells, which causes defects in muscle growth and regeneration. However, myotube-specific constitutive Notch activation actually improves regeneration in aged and dystrophic muscles (Table 2; Bi et al., 2016).

With regards to DMD treatment development, Notch signaling has been targeted in prior attempts at transplant-based therapeutic approaches. For instance, for engraftment to be successful, cells must be expanded *ex vivo* since a single donor muscle biopsy does not provide enough cells to meaningfully affect the muscle mass of a DMD patient (Parker et al., 2012). The way in which satellite cells are cultured meaningfully affects their effectiveness after transplant for the repair of dystrophic muscle (Parker et al., 2012; Parker and Tapscott, 2013). Studies have demonstrated that *ex vivo* expansion on tissue culture plates coated with Dll1-IgG fusion protein inhibits differentiation and increases levels of genes normally expressed in satellite cells, which leads to more effective engraftment and regeneration (Parker et al., 2012; Parker and Tapscott, 2013). Therefore, approaches incorporating components of the Notch pathway may be beneficial in transplant-based therapies.

Furthermore, therapeutic applications of the Notch pathway have been explored with regard to other topics in developmental and cancer biology (Zohorsky and Mequanint, 2021). Some studies have tried delivering soluble ligands in an attempt to activate Notch signaling, though these attempts have been largely unsuccessful (Zohorsky and Mequanint, 2021). Hence, the focus is being directed to the embedded and immobilized delivery of Notch ligands in order to facilitate activation of the endogenous pathway (Zohorsky and Mequanint, 2021). Thus far, Notch ligands have been immobilized to two-dimensional surfaces to examine the behavior of various cell types, including satellite cells (Parker et al., 2012; Safaee et al., 2017; Zohorsky and Mequanint, 2021). This idea has been employed in practice to respond to several maladies. For instance, one study found that the delivery of Jag1 to wounds was able to enhance wound healing (Chigurupati et al., 2007). Another found that delivery of Jag1-containing hydrogels inhibited myofibroblast differentiation in order to counteract cardiac fibrosis and expedite cardiac repair (Boopathy et al., 2015). Utilizing a different approach, another group found that implementing soluble Jag1 via stents could inhibit Notch signaling and subsequently prevent restenosis in vein grafts (Zhou et al., 2015). Hence, research thus far has demonstrated success in targeting the Notch pathway via multiple approaches in order to achieve varied goals, suggesting promise for targeting the Notch pathway in DMD treatments. It should be noted that the Notch pathway has been successfully targeted in cancer therapies as well, given the wealth of Notch research done through the lens of cancer biology and the implication of abnormal activation and expression of the Notch pathway in cancers, especially breast cancer and liver cancer (Zhang et al., 2019; Jia et al., 2021). Over the past 10 years, several new classes of drugs have emerged to therapeutically target Notch in cancer, acting to limit Notch signaling to reduce the pathway’s pro-oncogenic effects (Moore et al., 2020). Delivery of γ-secretase inhibitors such as DAPT has been effective, as well as receptor/ligand antibodies and Notch transcription complex inhibitors (Moore et al., 2020; Jia et al., 2021). A pro-inflammatory cytokine, IL-1β, is known to promote cell cycle progression of non-dystrophic myogenic cells but not DMD myogenic cells. Jag1, which is induced by IL-1β in DMD but not by normal cells, reduces the proliferation and differentiation of myogenic cells. Therefore, up-regulation of Jag1 by IL-1β plays a crucial role in the loss of muscle regeneration capacity of DMD muscles (Table 2; Nagata et al., 2017). Finally, inhibition of Notch and TGF-β promotes myogenic differentiation of human DMD-derived induced Pluripotent Stem Cells (iPSCs) (Table 2; Choi et al., 2012). With respect to muscular dystrophy treatment, the opposite approach would likely be taken, attempting to increase Notch activity rather than decrease it as is done in cancer treatments.

Finally, mutations in several components of the Notch pathway have been implicated in other various hereditary disorders, in addition to muscular dystrophies (Table 2). Missense mutations in Notch ligands *Jag1*, *Dll1*, and *Dll4* have been linked to Alagille syndrome I (ALGS), nonspecific neurodevelopmental disorders, and Adams-Oliver Syndrome 6 (AOS6), respectively (Coppens et al., 2021). CGG repeat expansions in the noncoding region of the *NOTCH2NLC* gene, a Notch inhibitor (Fiddes et al., 2018), can enhance Notch signaling. These expansions have been associated with several diseases, including multiple system atrophy (MSA), leukoencephalopathy, and forms of dementia including Alzheimer’s disease and frontotemporal dementia (Fiddes et al., 2018; Ogasawara et al., 2020). CGG repeats of *NOTCH2NLC* have also been implicated as a causative factor in neuronal intranuclear inclusion disease (NIID) and oculopharyngodistal myopathy (OPDM), both neurodegenerative diseases that involve progressive muscle weakness (Table 2; Ogasawara et al., 2020). Moreover, mutations at splice sites in different Notch receptors and ligands have also been shown to influence Notch signaling and have been implied in various pathologic phenotypes (Vargas-Franco et al., 2022). The splicing mechanism is somewhat unclear, though one study linked hnRNPL, a splicing regulator, to the Notch signaling pathway in several ways; overexpression of a partner of hnRNPL in zebrafish has been shown to destabilize the NICD and inhibit Notch signaling, studies in mice demonstrated that hnRNPL level is increased when Dll3 (a Notch inhibitory ligand) is lost, the fly homolog of hnRNPL (smooth) genetically modifies Notch, and hnRNPL downstream RNA targets include multiple components of the Notch pathway, including receptors Notch2 and Notch3 (Vargas-Franco et al., 2022). Though the specific mechanism remains unknown, possible splicing defects in Notch pathway components have been associated with many specific diseases. ALGS is normally caused by mutations in the *Jag1*, and heterozygous mutation at a splicing site just before exon 11 has been identified as a culprit (Zhu et al., 2021). Though much less frequently, ALGS is also caused by mutations in the *Notch2*, and a splice site mutation within the ankyrin repeats leads to decreased Notch signaling, playing a role in the pathology (Kamath et al., 2012). A variant in *Notch4* has been associated with schizophrenia, and recent analyses suggested that polymorphisms affecting the alternative splicing of *Notch4* may increase schizophrenia susceptibility (Shayevitz et al., 2012). Splice site mutations in *Notch3* have been linked to CASADIL (Cerebral Autosomal Dominant Arteriopathy with Subcortical Infarcts and Leukoencephalopathy) as a potential source of pathology (Lundkvist et al., 2005). A splice site variant of *Dll4*, which includes an in-frame insertion of 12 bp, has been associated with a variable neurodevelopmental phenotype (Fischer-Zirnsak et al., 2019). Lastly, studies in zebrafish have demonstrated that different splice variants of *deltaC*, a Notch ligand, cannot replace the function of one another during midline formation and somitogenesis (Mara et al., 2008).

## Other proteins associated with the Notch pathway: MEGF10 and POGLUT1

It should be noted that there are several interactions between the Notch pathway and other components in satellite cells, which work together to govern satellite cell proliferation. Multiple EGF-like domains 10 (MEGF10) is a transmembrane receptor expressed in both developing muscle satellite cells and myoblasts, which has exhibited marked similarity to Notch (Saha et al., 2017; Draper et al., 2019; Li et al., 2021). MEGF10 regulates myogenesis in conjunction with the Notch pathway, and MEGF10 deficiency displays several characteristics similar to Notch deficiency (Draper et al., 2019; Li et al., 2021). Biallelic loss of function of *MEGF10* causes MEGF10 myopathy or Early-onset myopathy, areflexia, respiratory distress and dysphagia (EMARDD), which involves areflexia, respiratory distress, and dysphagia (Table 2; Logan et al., 2011; Saha et al., 2017). A suggested cellular mechanism for disease in MEGF10 myopathy is slower proliferation and migration of satellite cells (Li et al., 2021). This leads to reduced MyoD expression and subsequent defects in myogenesis, which contributes to impaired skeletal muscle regeneration after injury seen in MEGF10 myopathy (Li et al., 2021). Several similarities and relationships have been identified between MEGF10 and the Notch pathway. First, sequence alignment of mammalian MEGF10 and its *drosophila* homolog, Drpr, have highlighted the conservation of domains that are characteristic of the Notch ligands (Draper et al., 2019). *Drpr* deficiency has been shown to lead to muscle abnormalities in flies. In addition, flies that overexpress mouse MEGF10 or fly Drpr display developmental arrest (Draper et al., 2019). In mice, there has been a suggestion for interaction between MEGF10 and the Notch pathway in regulating myogenesis (Table 2; Saha et al., 2017). Further, it has been shown that knockdown of *Megf10* results in downregulation of *Notch1*, *Notch2*, *Notch3*, and *Hes1*, hence suggesting that the extracellular domain of MEGF10 may act as a ligand to activate Notch signaling (Holterman et al., 2007). Also, MEGF10 myopathy involves impaired tyrosine phosphorylation, which causes impaired interaction between MEGF10 and the Notch signaling pathway (Li et al., 2021). Similarly to Notch, deficits seen with MEGF10 myopathy have suggested MEGF10 as a target for potential therapies for muscle diseases; rescuing MEGF10 may have therapeutic implications with regard to ameliorating impaired muscle regeneration (Li et al., 2021).

Protein glycosylation is one of the major regulatory mechanisms of the signaling pathway (Pandey et al., 2021). The extracellular domain of the Notch receptor is modified with *O-*fucose and *O*-glucose glycans, and this glycosylation is crucial for the activity of the pathway (Takeuchi et al., 2018). POGLUT1 is involved in the post-translational modification and function of Notch receptors and ligands by reducing *O*-glucosyltransferase activity on Notch, receptors and ligands and missense mutations in *POGLUT1* were identified in a family with autosomal recessive limb-girdle muscular dystrophy (LGMD) (Table 2; Servián-Morilla et al., 2016). Primary myoblasts from patients with *POGLUT1* mutations demonstrate slow proliferation, a decreased pool of quiescent satellite cells and a decreased Notch signaling in their muscle tissues (Table 1; Servián-Morilla et al., 2016). Studies have also identified potential interactions between POGLUT1 and Jag1, though the biological relevance is still unclear (Pandey et al., 2021).

α-Dystroglycan is a glycosylated protein and a key component of the dystrophin-glycoprotein complex (DCG), as it is essential for normal basement membrane development and muscle maintenance (Servián-Morilla et al., 2016). Its extracellular subunit is modified with *O*-linked glycans, which is necessary for the proliferation of satellite cells. One study focused on ribitol-phosphate modification, which is necessary for functional maturation of α-dystroglycan (Tokuoka et al., 2022). Cytidine-diphosphate (CDP-ribitol) is a donor substrate for ribitol-phosphate modification , and proof-of-concept work indicated that supplementation therapy with CDP-ribitol could accelerate development of therapeutic agents for diseases involving glycosylation defects, including DMD (Tokuoka et al., 2022). These results suggest that modulations of Notch protein and pathway may be a promising therapeutic target for muscular dystrophies.

## Conclusion

Due to their prevalence and severity, it is imperative that we continue to search for effective therapies for muscular dystrophies, including DMD. The pathogenesis of DMD and other muscular dystrophies has been well-characterized, and there has been a convincing indication that depletion of the satellite cell pool is implicated in the degeneration that defines the muscular dystrophy phenotype. By targeting the Notch signaling pathway, therapies have the potential to selectively increase the proliferation of satellite cells, thereby hopefully ameliorating the muscular dystrophy phenotype and dramatically improving the quality of life for those living with the condition. Prior work has demonstrated some success with satellite cell-transfer therapies, as well as with targeted upregulation of certain components of the Notch signaling pathway, including the ligand Jag1. Further research is necessary for the optimization of these therapies and the exploration of different manners in which the power of the Notch signaling pathway can be harnessed in order to combat muscular dystrophies.
